# Psychotic symptoms with and without a primary psychotic disorder in children requiring inpatient mental health admission

**DOI:** 10.1192/j.eurpsy.2024.23

**Published:** 2024-03-05

**Authors:** Nefeli Anagnostopoulou, Efstathios Papachristou, Hayley Galitzer, Anca Alba, Jorge Gaete, Danai Dima, Maria Rogdaki, Gonzalo Salazar de Pablo, Marinos Kyriakopoulos

**Affiliations:** 1 South London and Maudsley NHS Foundation Trust, London, UK; 2 UCL Institute of Education, Department of Psychology & Human Development, London, UK; 3 Division of Internal Medicine, Stanford University School of Medicine, Stanford, CA, USA; 4 Counselling London and Mental Health Support Service, King’s College London, London, UK; 5 Faculty of Education, Universidad de los Andes, Santiago, Chile; 6 Millennium Nucleus to Improve the Mental Health of Adolescents and Youths (Imhay), Santiago, Chile; 7 Department of Psychology, School of Health and Psychological Sciences, City, University of London, London, UK; 8 Department of Neuroimaging, Institute of Psychiatry, Psychology and Neuroscience, King’s College London, London, UK; 9 Department of Child and Adolescent Psychiatry, Institute of Psychiatry, Psychology and Neuroscience, King’s College London, London, UK; 10 Department of Psychosis Studies, Institute of Psychiatry, Psychology and Neuroscience, King’s College London, London, UK; 11 Institute of Psychiatry and Mental Health, Department of Child and Adolescent Psychiatry, Hospital General Universitario Gregorio Marañón School of Medicine, Universidad Complutense, Instituto de Investigación Sanitaria Gregorio Marañón (IiSGM), CIBERSAM, Madrid, Spain; 12 1st Department of Psychiatry, National and Kapodistrian University of Athens, Athens, Greece

**Keywords:** childhood-onset schizophrenia, children, inpatient, psychotic disorder, psychotic symptoms

## Abstract

Psychotic symptoms are relatively common in children and adolescents attending mental health services. On most occasions, their presence is not associated with a primary psychotic disorder, and their clinical significance remains understudied. No studies to date have evaluated the prevalence and clinical correlates of psychotic symptoms in children requiring inpatient mental health treatment. All children aged 6 to 12 years admitted to an inpatient children’s unit over a 9-year period were included in this naturalistic study. Diagnosis at discharge, length of admission, functional impairment, and medication use were recorded. Children with psychotic symptoms without a childhood-onset schizophrenia spectrum disorder (COSS) were compared with children with COSS and children without psychotic symptoms using Chi-square and linear regressions. A total of 211 children were admitted during this period with 62.4% experiencing psychotic symptoms. The most common diagnosis in the sample was autism spectrum disorder (53.1%). Psychotic symptoms were not more prevalent in any diagnosis except for COSS (100%) and intellectual disability (81.8%). Psychotic symptoms were associated with longer admissions and antipsychotic medication use. The mean length of admission of children with psychotic symptoms without COSS seems to lie in between that of children without psychotic symptoms and that of children with COSS. We concluded that psychotic symptoms in children admitted to the hospital may be a marker of severity. Screening for such symptoms may have implications for treatment and could potentially contribute to identifying more effective targeted interventions and reducing overall morbidity.

## Introduction

Psychotic symptoms, including hallucinations and delusions, are the main characteristics of psychotic disorders. However, several lines of evidence suggest that their presence can be detected in many other mental health conditions [1, 2]. Prevalence estimates for schizophrenia across the lifespan range from 0.4 to 1% in the general population depending on the examined country [3, 4] with these rising to almost 3.5%, when all psychotic disorders, including severe depression with psychotic features, bipolar disorder type I and drug-induced psychosis [5] are grouped together. However, the prevalence of psychotic symptoms in the general population appears to be considerably higher, reaching, in one study, even the rate of 31.4% [6]. A degree of variability in the identified prevalence of psychotic symptoms is largely related to the definition of what constitutes a psychotic or “psychotic-like” symptom. Nevertheless, the frequency at which unusual experiences are seen in individuals without a psychotic disorder is remarkable. In the most comprehensive review of the literature, the prevalence rate for sub-clinical psychotic experiences in community samples aged 16 to 75 was estimated at 7.2% [7]. The high prevalence of psychotic symptoms in the general population, their association with different mental disorders, and their homotypic discontinuity over time [8], warrant further research on psychotic symptoms in clinical samples. Surprisingly, there is very limited evidence on the prevalence of psychotic symptoms in people receiving treatment for a mental disorder. Most prevalence studies on clinical samples include older adolescents or adults [9] or focus on the cooccurrence of schizophrenia spectrum disorders with other clinical correlates rather than on the prevalence of psychotic symptoms [10].

The clinical evaluation of the significance of psychotic symptoms may be even more complicated in younger populations. The prevalence of at least one psychotic symptom in children in the general population was 17%, while the prevalence for adolescents rounded up to 7.5% [11]. In a more recent review and meta-analysis focusing on auditory hallucinations across the lifespan, their mean prevalence rates were 12.7% in children, 12.4% in adolescents, and 5.8% in adults aged 18 to 60 years [12]. This significant reduction in the prevalence of psychotic symptoms from childhood to adulthood suggests that these symptoms may emerge as part of the developmental trajectory of some people without being specifically linked to psychosis. Children and young people attending community mental health clinics may experience psychotic symptoms even more commonly, with such symptoms having been reported in 43–97.7% of these samples [13–16]. Psychotic symptoms may also represent markers of clinical severity as suggested by their association with the number of comorbid disorders in these children and young people [17]. Nevertheless, their high prevalence both in population and in clinical studies support their lack of specificity in terms of risk for psychosis.

Psychotic symptoms and disorders can significantly impact an individual’s cognitive and social functioning [18], relationships, and quality of life [19]. This is especially important for children and adolescents as earlier appearance of symptoms has been linked with more severe difficulties in later life [20,21]. Transition to a diagnosable psychotic disorder in these children and young people is uncommon; even among those meeting criteria for clinically high risk for psychosis, 9.5% transition to psychosis at 1 year, 12.1% at 2 years, and 16.1% at >5 years which reduces further to 3.9% at 1 year, 11.6% at 2 years, and 14.3% at >5 years when only studies with a low risk of bias were considered in a recent meta-analysis [22]. Furthermore, the management of children and young people presenting with psychotic symptoms not meeting the criteria for a psychotic disorder can be challenging as there is no evidence of effective intervention to prevent the emergence of psychosis in this age group [23].

Our study aims to examine the prevalence and clinical correlates of psychotic symptoms in children aged 6 to 12 years requiring treatment in an inpatient mental health unit. This is likely to enhance our understanding of how psychotic symptoms may affect younger children across a range of severe mental health presentations requiring higher level of input and may assist in the exploration of the nature of these clinical symptoms. To the authors’ knowledge, this is the first study investigating this, with the use of a unique sample of pre-adolescent children with severe mental health needs. The examination of psychotic symptoms in this sample of children and their associations with different psychiatric diagnoses, such as depressive, anxiety, or neurodevelopmental disorders, is likely to guide diagnostic approaches that are useful in clinical practice and lead to a better understanding of the significance of psychotic symptoms across diagnoses. Our hypothesis was that there would be a high prevalence of psychotic symptoms in our sample and that psychotic symptoms would be present across different diagnoses. We also hypothesized that psychosis and psychotic symptoms would be associated in a dose-dependent way (psychotic disorder > psychotic symptoms > no psychotic symptoms) with clinical severity as inferred by longer hospital admissions and more frequent use of medication.

## Methods

### Participants

This “Strengthening the Reporting of Observational Studies in Epidemiology” (STROBE) [24] compliant investigation included all children needing inpatient mental health treatment in a UK National Children’s Unit from January 2009 to June 2018. As per the unit’s treatment inclusion criteria, children were between the ages of 6 and 12 years with suspicion or a diagnosis of a mental health disorder. No exclusion criteria were applied, as the goal was to examine the whole of the inpatient population.

### Study design

We followed the protocol of a retrospective naturalistic clinical prevalence study. Data related to routine clinical care were extracted from the patients’ electronic notes. These were based on assessments children had, including clinical history, clinical observations, and direct interviews. The lead researcher assessed each child’s a) mental health examinations, b) clinical notes, c) psychological session notes, d) admission and discharge summaries, as well as e) multidisciplinary meeting minutes to extract the relevant information to the present study. This investigation was part of a wider service evaluation project of routine clinical care approved by the South London and Maudsley Child and Adolescent Mental Health Services Clinical Academic Group Clinical Governance/Audit Committee, UK. All subsequent analyses were conducted on anonymized data.

### Procedure

Information was extracted from the patients’ electronic records with the assistance of a mining algorithm identifying words that indicated the presence of psychotic symptoms. Those were “unusual experiences” and “unusual beliefs,” “hallucination” and “hallucinations,” “delusion” and “delusions,” “voices,” “visions,” “paranoid” and “paranoia,” and “suspicion” and “suspicious”. Once these words were identified, the records were inspected by one of the authors (NA), and in case of ambiguity, were discussed between two of the authors (NA and MK) to ensure that the child experienced these symptoms and confirm the clinical impression at the time of the symptom. Diagnosis at discharge, length of admission in days, Children’s Global Assessment Scale scores (CGAS) [25] on admission and at discharge, and whether children were taking or not (yes/no) medication on admission and at discharge, and antipsychotics at any point and at discharge were also extracted for each patient. Diagnoses were made in accordance with the Multiaxial ICD-10 classification of child and adolescent psychiatric disorders [26] and confirmed using the ICD-10 diagnostic criteria for research [27]. The diagnoses specifically examined in relation to psychotic symptoms in this study included schizophrenia spectrum disorders (ICD-10 F20–F29), depressive disorders (ICD-10 F32–F33), anxiety disorders (ICD-10 F40–F41), obsessive-compulsive disorder (OCD; ICD-10 F42), eating disorders (ICD-10 F50), intellectual disability (ID; ICD-10 F70–F79), pervasive developmental disorders (autism spectrum disorders, ASD; ICD-10 F84), and hyperkinetic disorders (attention deficit hyperactivity disorder, ADHD; ICD-10 F90). The total number of diagnoses for each child was also recorded.

### Data analysis

All data were transformed into quantitative data for analysis purposes. We then ran descriptive statistics of the sample’s sociodemographic and clinical characteristics. Next, Chi-square tests were performed to examine differences in the rates of any psychotropic and antipsychotic medication use on admission and discharge between children without psychotic symptoms and those with psychotic symptoms but without child-onset schizophrenia spectrum disorders (COSS).

Finally, we ran a series of linear regression analyses to examine whether children with psychotic symptoms without COSS differed from children with COSS or those without psychotic symptoms in relation to their duration of treatment, before and after adjustments for confounding factors, including children’s sex, age, number of diagnoses, medication at admission, and CGAS scores at admission. Three dummy variables were created for each of the three groups and the one left out served as the reference group for the respective model. In the first regression model (model A), we compared whether the three groups differed in terms of the duration of treatment without covariates. A second regression model (model B) examined if the three groups differed in duration of treatment, but we adjusted the models for sex and age at admission (in months). Finally, the third model (model C) included adjustments for sex, age, number of diagnoses, medication at admission, and CGAS scores at admission. All analyses were run using a maximum likelihood estimator with robust standard errors (MLR) to account for the skewed distribution of variables. We considered as significant estimates with *p*-values <0.05.

Because the group of children with psychosis was under-represented in the sample (*n* = 20), we replicated the models using a Bayesian estimator as a sensitivity analysis. Bayesian approaches offer an intuitive and viable alternative in situations where the sample size is small. In the absence of previous literature on this topic, we employed non-informative priors and treated the Bayesian estimation merely as a computational tool for getting estimates analogous to the ones obtained using MLR. For these models, the Markov Chain Monte Carlo (MCMC) algorithm based on the Gibbs sampler was used, as implemented in Mplus 7.4 [28]. Model fit of the models using the Bayesian estimator was assessed using the results of the Chi-square test, which compares the distribution of the replicated Chi-square value to the observed Chi-square value (non-significant differences provide evidence in favor of the model’s validity and reliability).

Analyses were run in SPSS Version 28 and Mplus 7.4 [28].

## Results

### Participants

Two-hundred and eleven children’s records were identified. The mean age of the patients was 129.7 months (approximately 11 years) and there was a roughly even sex ratio (55.0% male). The most common diagnosis in this sample was ASD (53.1%), followed by anxiety disorders (34.1%), ADHD (27.0%) and depressive disorders (16.6%). A relatively small proportion (9.5%) had COSS (Table 1). Diagnoses were considered independently and were non-exclusionary as many children presented with more than one mental health disorder.

**Table 1.** Demographic/clinical characteristics and outcomes for the whole sample

Variables	*N* = 211
Age of admission in months (SD)	129.7 (20.1)
Female sex (%)	95 (45)
Medication at Admission (%)	114 (54)
Mean CGAS score on admission (SD)	27.2 (13.2)
Mean CGAS score at discharge (SD)	56.7 (16)
Diagnoses (%)	
ASD	112 (53.1)
Anxiety disorder	72 (34.1)
ADHD	57 (27)
Depressive disorder	35 (16.6)
ID	33 (15.6)
OCD	32 (15.2)
COSS	20 (9.5)
Eating disorder	17 (8.1)
Psychotic symptoms for each diagnosis^#^ (%)	
COSS	100**
ID	81.8*
Depressive disorder	65.7
Anxiety disorder	65.3
ASD	64.3
ADHD	63.2
OCD	53.1
Eating disorder	52.9
Outcomes	
Length of Admission in days (SD)	141.4 (91.9)
Medication at discharge (%)	164 (77.7)
Antipsychotics at discharge (%)	96 (45.4)
Antipsychotics at any point (%)	123 (58.3)

Abbreviations: SD, standard deviation; ASD, autism spectrum disorders; ADHD, attention deficit hyperactivity disorder; ID, intellectual disability; OCD, obsessive-compulsive disorder; COSS, childhood-onset schizophrenia spectrum disorders; CGAS, Children Global Assessment Scale.

Medication: Any medication, including antipsychotics, antidepressants, mood stabilizers, stimulants, atomoxetine, and alpha 2 agonists.

# *p*-values refer to the comparison in relation to the presence of psychotic symptoms between children who had the diagnosis and children who did not have it, using Pearson’s Chi-square test.

* *p* < 0.05.

** *p* < 0.001.

### Prevalence of psychotic symptoms

A total of 132 children (62.6%) experienced psychotic symptoms. Psychotic symptoms were almost exclusively identified after the children were directly asked about them or through clinical observations. Hallucinations (56.9%) seem to be more prevalent than delusions (34.6%). Psychotic symptoms were frequently identified in most diagnoses (Table 1). In comparisons of children who had a specific diagnosis with those who did not have this diagnosis, psychotic symptoms did not seem to be more prevalent in any diagnoses other than COSS, with the exception of ID in our sample (*r* (211) = 0.171, *p* = 0.013).

### Medication use

More than half of the sample (54.0%), including children with COSS, were already taking psychotropic medication at the point of admission, and by the point of discharge, this percentage had increased to 77.7%, with 45.7% of all children taking antipsychotic medication. The latter compares favorably to the percentage of children who had been taking an antipsychotic at any point in their treatment, even before hospital admission (58.3%), and shows that the admission was associated with a reduction in antipsychotic use (Pearson’s *χ* ^2^ = 125.08, *df* = 1, *p* < 0.001). All children with COSS were taking antipsychotic medication at discharge. The mean length of stay in the unit was around 4.5 months (*M* = 141.4 days, *SD* = 91.9) (Table 1). Compared to children without psychotic symptoms, children with psychotic symptoms without COSS were more likely to be taking antipsychotic medication at any point (Pearson’s *χ* ^2^ = 13.107, *df* = 1, *p* < 0.001) and at discharge (Pearson’s *χ* ^2^ = 6.036, *df* = 1, *p* = 0.014) but not any psychotropic medication on admission (Pearson’s *χ* ^2^ = 3.408, *df* = 1, *p* = 0.065) or at discharge (Pearson’s *χ* ^2^ = 2.805, *df* = 1, *p* = 0.094).

### Presence of psychotic symptoms as a predictor of the duration of treatment

The results of the linear regression models comparing the three groups (non-COSS children with psychotic symptoms/children with COSS/children without psychotic symptoms) in relation to the duration of their treatment are presented in Table 2. The results of the unadjusted model (model A) suggest that non-psychotic children with psychotic symptoms were treated on average 28 days longer than those without psychotic symptoms (*b* = −28.15, *p* = 0.02). This association was attenuated yet remained statistically significant after adjustments for sex and age at admission (model B; *b* = −27.93, *p* = 0.02) and in the fully adjusted model (model C; *b* = −23.93, *p* = 0.05). Results of the fully adjusted models additionally suggested that none of the covariates, that is, sex, age at admission, number of diagnoses, receiving medication at admission, or CGAS scores at admission, were significantly related to the duration of treatment.

**Table 2.** Crude and adjusted unstandardized multiple linear regression coefficients (SE) for the duration of admission (in days) in the analytic sample (*N* = 211)

	Model A	Model B	Model C
*Intercept*	147.46 (8.90)**	159.32 (39.28)**	133.14 (30.52)**
*Group 1*: Any diagnosis (excluding COSS) with psychotic symptoms	Reference	Reference	Reference
(Reference; *N* = 112; 53.1%)			
*Group 2: COSS* (*N* = 20; 9.5%)	47.20 (28.74)	50.16 (29.31)	52.02 (30.52)
*Group 3:* No psychotic symptoms (*N* = 79; 37.4%)	−28.15 (11.86)*	−27.93 (11.72)*	−23.93 (12.02)*
Sex, female (*N* = 95; 45.0%)	—	−13.79 (13.05)	−9.86 (12.95)
Age at admission (months)	—	.06 (.31)	.03 (.31)
Number of diagnoses	—	—	4.07 (6.20)
Medication at admission (*N* = 114–54.0%)	—	—	21.90 (12.48)
CGAS at admission	—	—	.08 (.46)

Abbrevaitions: COSS, Childhood-onset schizophrenia spectrum disorders; CGAS, Children Global Assessment Scale.

* *p* < 0.05.

** *p* < .0.01.

Finally, we re-ran model C after changing the reference category to children with COSS (results not shown in Table 2). The results of this revised fully adjusted model suggested that children with COSS were treated on average 75 days longer than children without psychotic symptoms (*b* = −75.94, SE = 29.35, *p* = 0.01) and 52 days longer than non-psychotic children with psychotic symptoms, albeit the latter finding did not reach statistical significance (*b* = −52.02, SE = 30.52, *p* = 0.09).

### Bias analysis

Considering the small overall sample size and, in particular, the group of children with psychosis (*n* = 20), we re-ran the fully adjusted models (model C) using a Bayesian estimator to assess the robustness of the previously presented regression estimates. The model showed good fit to the data (Chi-square, 95% CI:11.29–10.28, *p* = 0.33) and confirmed the findings obtained by the classical regression models. The results suggest that non-COSS children with psychotic symptoms were treated on average 22 days longer than children without psychotic symptoms (*b* = −22.54, posterior SD = 12.83, *p* = 0.02) and 55 days less than children with COSS (*b* = 54.88, posterior SD = 23.10, *p* < 0.001). Moreover, compared to children with COSS, those without psychotic symptoms were treated on average 75 days less (*b* = −75.07, posterior SD = 20.09, *p* < 0.001), and those with psychotic symptoms but without COSS were treated on average 49 days less (*b* = −49.92, posterior SD = 23.02, *p* = 0.02).

## Discussion

To the authors’ knowledge, this is the first study to assess the prevalence of psychotic symptoms in a unique inpatient clinical sample of children and their associations with different mental health disorders. Psychotic symptoms were very prevalent in this population, affecting 62.6% of the sample and similarly spread across several diagnoses. While no other studies, including only children treated in an inpatient mental health unit, could be identified, our findings are in line with the literature on community mental health services for children and adolescents, suggesting a high prevalence of psychotic symptoms in these clinical samples [13–16]. Notably, Gin and colleagues previously showed that 60% of children and adolescents in the community would self-report psychotic symptoms associated with distress or adverse functional impact during their initial assessment [14]. Such symptoms were spread across diagnoses in community clinical populations as well [13,15,16].

In our sample, psychotic symptoms did not seem to be associated with any specific diagnosis with the exception of COSS, as expected, and ID. Overall, this finding seems to support the notion of a psychosis continuum where psychotic symptoms are present across psychiatric diagnoses and not limited to psychotic disorders [29]. In relation to ID, while there is a possibility that psychotic symptoms may be more common in children with these conditions in need of mental health inpatient treatment, this finding should be interpreted with caution. In the UK, children with ID are receiving a wide range of community and specialist education packages, which may make it more likely for mental health difficulties to be successfully managed outside hospitals. As a result, this association may be related to selection bias in that admission to hospital for children with ID is organized for those who cannot be managed with this higher level of care and are therefore presenting with more severe psychopathology, including psychotic symptoms.

The high prevalence of psychotic symptoms in our population, combined with the fact that they were spread across diagnoses, has several clinical implications. First, it highlights the importance of routinely screening for psychotic symptoms during initial assessments. As sometimes people who experience them are reluctant to reveal so by themselves [30], incorporating relevant questions as part of the clinical interview or a routine screening tool would be essential. Indeed, in our study, psychotic symptoms were either reported after the children were directly asked about them or through clinical observations. Similarly, in the study by Gin and colleagues [14], the high percentage of reported psychotic symptoms in community mental health services for children and adolescents came after four services started piloting screening for psychotic symptoms during initial assessments. Second, our results showed that psychotic symptoms are associated with longer hospital admissions and more frequent antipsychotic medication use. If that is the case, the presence of psychotic symptoms at such a young age may also be adversely associated with the children’s trajectory of mental health difficulties. This assumption is in line with previous research identifying that children experiencing psychotic symptoms have a high risk of experiencing or developing a wide range of psychopathology, including but not limited to psychotic disorders [22,31–34]. Unfortunately, the nature of psychotic symptoms is still not well understood. Although they may represent an aspect of brain maturation that, on most occasions, is part of non-highly-atypical development, accumulating evidence on their implications, such as those mentioned above, may suggest pathophysiological brain processes that lead to diverse brain trajectories giving rise to such phenomenology. More research is needed in order to further understand the nature of psychotic symptoms and evaluate clinical outcomes in the group of children experiencing them.

In addition, there is an emerging literature on psychotic symptoms being related to the severity of mental health difficulties. Psychotic symptoms in childhood are reported to be mostly transient, while in adolescence, these are more likely to be associated with psychopathology and with severe, multiple diagnoses [12,17]. There is also some evidence to suggest that children and adolescents with psychotic disorders are more prone to re-admissions [35] and longer hospital stays [36]. However, most clinical studies have focused on adolescent youths rather than children. Our study adds to this body of literature on possible clinical implications of psychotic symptoms in children with mental health needs, suggesting that their presence also marks disorder severity in this age group. Their potential link with longer admissions and higher antipsychotic medication use, as our study suggests, highlights the need for changes in clinical practice, potentially incorporating more specific psychological therapies, for example, family interventions, cognitive behavioral therapy, or medications associated with less side effects. Although psychological therapies have limited use in reducing psychotic symptoms in early-onset psychotic disorders, they may positively affect psychosocial functioning [37], and possibly have the potential to reduce the length of inpatient admissions. In addition, unusual experiences in children and young people with non-psychotic disorders may respond better to psychological treatment, for example, with cognitive behavioral therapy [38], making the screening for such experiences relevant to their treatment and potentially contributing to improved clinical outcomes.

### Strengths and limitations

The main strength of the current study is related to the inclusion of the whole sample of a unique population of children with severe mental health difficulties admitted to an inpatient unit over a 9-year period. It captured the presence of psychotic symptoms through different means, including self-report, comprehensive interviews, and clinical observations as part of the children’s clinical care. Its limitations include the use of outcome measures related to routine clinical care, the lack of prospective data with the use of relevant questionnaires, and the inability to potentially capture additional factors that may have affected the length of admission or medication use, like social or educational factors. It is possible that these latter factors may apply differentially in children presenting with COSS or psychotic symptoms. Finally, our study could not characterize children in terms of clinical high risk for psychosis beyond the clinical implications of them experiencing psychotic symptoms, which is an important topic of future research.

## Conclusion

In conclusion, our study suggests that the presence of psychotic symptoms in children requiring inpatient mental health treatment has clinical implications related to the severity of the children’s presentation as inferred by longer admissions and antipsychotic medication use. The mean length of admission of children with psychotic symptoms without COSS seems to lie in between that of children without psychotic symptoms and that of children with COSS, which is suggestive of psychotic symptoms being a marker of severity in a dose-related manner. Specific evaluation of psychotic symptoms in children receiving mental health treatment in an inpatient setting, and potentially more broadly, is likely to allow targeted psychological interventions that may reduce antipsychotic medication use and overall morbidity.
